# The pyrochlore-type molybdate Pr_1.37_Ca_0.63_Mo_2_O_7_

**DOI:** 10.1107/S1600536808015328

**Published:** 2008-05-24

**Authors:** P. Gall, P. Gougeon

**Affiliations:** a Unité Sciences Chimiques de Rennes, UMR CNRS No. 6226, Université de Rennes I–INSA Rennes, Campus de Beaulieu, 35042 Rennes CEDEX, France

## Abstract

Praseodymium calcium dimolybdenum hepta­oxide, Pr_1.37_Ca_0.63_Mo_2_O_7_, crystallizes in the cubic pyrochlore-type structure. In the crystal structure, MoO_6_ octa­hedra are linked together by common corners, forming a three-dimensional [Mo_2_O_6_] network. The Pr and Ca atoms and the remaining O atoms are located in the voids of the [Mo_2_O_6_] network. The Pr and Ca atoms are distributed statistically over the same 16c crystallographic position with site-occupancy factors of 0.684 (3) and 0.316 (3), respectively. They are surrounded by eight O atoms forming a ditrigonal scalenohedron. All atoms lie on special positions. The (Pr, Ca) and Mo atoms are, respectively in the 16*c* and 16*d* positions with 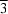 *m* symmetry, and the O atoms in the 48*f* or 8*a* positions with *mm* or 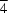
3*m* site symmetry, respectively.

## Related literature

For related literature, see: Hubert (1974 ▶); Subramanian *et al.* (1983 ▶); American Chemical Society (2007 ▶); Gougeon *et al.* (2003 ▶); Kerihuel & Gougeon (1995 ▶).

## Experimental

### 

#### Crystal data

Pr_1.37_Ca_0.63_Mo_2_O_7_
*M* *_r_* = 522.18
Cubic, 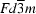

*a* = 10.4329 (3) Å
*V* = 1135.57 (6) Å^3^
*Z* = 8
Mo *K*α radiation
μ = 16.45 mm^−1^
*T* = 293 (2) K
0.16 × 0.14 × 0.12 mm

#### Data collection

Nonius KappaCCD diffractometer
Absorption correction: analytical (de Meulenaer & Tompa, 1965 ▶) *T*_min_ = 0.093, *T*_max_ = 0.125
771 measured reflections
266 independent reflections
167 reflections with *I* > 2σ(*I*)
*R*_int_ = 0.053

#### Refinement

*R*[*F* ^2^ > 2σ(*F* ^2^)] = 0.027
*wR*(*F* ^2^) = 0.086
*S* = 1.11
266 reflections
12 parameters
Δρ_max_ = 2.51 e Å^−3^
Δρ_min_ = −1.55 e Å^−3^

### 

Data collection: *COLLECT* (Nonius, 1998 ▶); cell refinement: *COLLECT*; data reduction: *EVALCCD* (Duisenberg *et al.*, 2003 ▶); program(s) used to solve structure: *SIR97* (Altomare *et al*., 1999 ▶); program(s) used to refine structure: *SHELXL97* (Sheldrick, 2008 ▶); molecular graphics: *DIAMOND* (Brandenburg, 2001 ▶); software used to prepare material for publication: *SHELXL97*.

## Supplementary Material

Crystal structure: contains datablocks global, I. DOI: 10.1107/S1600536808015328/pk2092sup1.cif

Structure factors: contains datablocks I. DOI: 10.1107/S1600536808015328/pk2092Isup2.hkl

Additional supplementary materials:  crystallographic information; 3D view; checkCIF report

## Figures and Tables

**Fig. 1.**
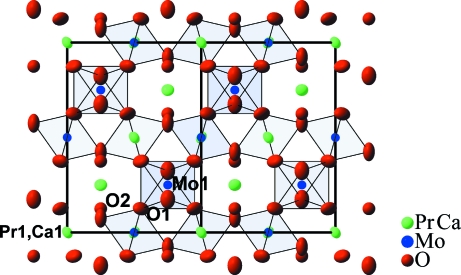
: View of Pr1.37Ca0.63Mo2O7 along the [110] direction. Displacement ellipsoids are drawn at the 97% probability level.

## supplementary crystallographic information

### Comment

An attempt to synthesize PrCaMo_16_O_28_, a compound with the PrMo_8_O_14_ type structure (Kerihuel & Gougeon, 1995), was unsuccessful, resulting in a multiphase product.  However, the formation of the new compound, Pr_1.37_Ca_0.63_Mo_2_O_7_ was achieved. A survey of the literature related to the rare earth molybdates *R*_2_Mo_2_O_7_ with the database SciFinder Scholar (American Chemical Society, 2007) shows that these compounds only form for the rare-earths from Nd to Lu. To our knowledge, no quaternary molybdate pyrochlore has thus far been reported.

### Experimental

Single crystals of Pr_1.37_Ca_0.63_Mo_2_O_7_ were prepared from a mixture of Pr_6_O_11_ (Rhone Poulenc, 99.99%), CaMoO_4_, MoO_3_ (Cerac, 99.95%) and Mo (Plansee, 99.9999%) with the nominal composition PrCaMo_16_O_28_. Before use, Mo powder was reduced under a flow of H_2_ gas at 1273 K for ten hours in order to eliminate any trace of oxygen. CaMoO_4_ was prepared by heating a stoichiometric mixture of CaCO_3_ and MoO_3_ in an open porcelain crucible at 1073 K for 24 h. The initial mixture (*ca* 5 g) was cold pressed and loaded into a molybdenum crucible, which was sealed under a low argon pressure using an arc welding system. The charge was heated at a rate of 300 K/h up to 2223 K, and the temperature was held for 5 min., then cooled at 100 K/h to 1373 K and finally furnace cooled. The final product was multiphasic with Pr_1.37_Ca_0.63_Mo_2_O_7_ and Pr_1 - _*_x_*Ca*_x_*Mo_10_O_16_, isomorphous with the RMo_5_O_8_ compounds (*R* = La to Gd; Gougeon *et al.*, 2003), as predominant phases.  The crystals thus obtained were of irregular shape.

### Refinement

The structure was solved by direct methods using *SIR97* (Altomare *et al.*,  1999). The second setting, with the origin at 3 m of the Fd3m space group, was chosen. Initial refinement with full occupancy for the Pr1 site resulted in an *R* factor of about 0.30. Refinement of the site-occupancy factor of the Pr1 atoms lowered the *R* factor to 0.0274 with an occupation factor of 0.74. As qualitative microanalyses using a Jeol JSM-35 CF scanning electron microscope equipped with a Tracor energy-dispersive-type X-ray spectrometer indicated the presence of calcium in the crystals, we surmised that the deficiency observed on the Pr1 site resulted from the presence of calcium. Refinements taking into account an occupation of the deficient Pr1 site simultaneously by Pr and Ca atoms with no constraint on the site-occupancy factors of the Pr1 and Ca1 atoms led to an over-occupation of the 16 d position. Consequently, the sum of the site occupancy factors was constrained to unity, and the ADPs of the Pr1 and Ca1 atoms were constrained to be equal. Refinement of the occupancy factor of the O2 atom in 8a position which frequently exhibits partial or total deficiency, indicates full occupation of this position.

### Crystal data

**Table:**

Pr_1.37_Ca_0.63_Mo_2_O_7_	*Z* = 8
*M**_r_* = 522.18	*F*_000_ = 1867.4
Cubic, *F**d*3*m*	*D*_x_ = 6.109 Mg m^−^^3^
Hall symbol: -F 4vw 2vw	Mo *K*α radiation λ = 0.71070 Å
*a* = 10.4329 (3) Å	Cell parameters from 1172 reflections
*b* = 10.4329 (3) Å	θ = 3.4–45.3º
*c* = 10.4329 (3) Å	µ = 16.45 mm^−^^1^
α = 90º	*T* = 293 (2) K
β = 90º	Irregular block, black
γ = 90º	0.16 × 0.14 × 0.12 mm
*V* = 1135.57 (6) Å^3^	

### Data collection

**Table:**

Nonius KappaCCD diffractometer	266 independent reflections
Radiation source: fine-focus sealed tube	167 reflections with *I* > 2σ(*I*)
Monochromator: horizontally mounted graphite crystal	*R*_int_ = 0.053
Detector resolution: 9 pixels mm^-1^	θ_max_ = 45.3º
*T* = 293(2) K	θ_min_ = 3.4º
φ scans (κ = 0) + additional ω scans	*h* = 1→20
Absorption correction: analytical(de Meulenaer & Tompa, 1965)	*k* = 0→14
*T*_min_ = 0.093, *T*_max_ = 0.125	*l* = 0→13
771 measured reflections	

### Refinement

**Table:**

Refinement on *F*^2^	Secondary atom site location: difference Fourier map
Least-squares matrix: full	*w* = 1/[σ^2^(*F*_o_^2^) + (0.0207*P*)^2^ + 4.885*P*] where *P* = (*F*_o_^2^ + 2*F*_c_^2^)/3
*R*[*F*^2^ > 2σ(*F*^2^)] = 0.027	(Δ/σ)_max_ < 0.001
*wR*(*F*^2^) = 0.086	Δρ_max_ = 2.51 e Å^−^^3^
*S* = 1.12	Δρ_min_ = −1.55 e Å^−^^3^
266 reflections	Extinction correction: SHELXL97 (Sheldrick, 2008), Fc^*^=kFc[1+0.001xFc^2^λ^3^/sin(2θ)]^-1/4^
12 parameters	Extinction coefficient: 0.00256 (19)
Primary atom site location: structure-invariant direct methods	

### Special details

**Table:**

Geometry. All e.s.d.'s (except the e.s.d. in the dihedral angle between two l.s. planes) are estimated using the full covariance matrix. The cell e.s.d.'s are taken into account individually in the estimation of e.s.d.'s in distances, angles and torsion angles; correlations between e.s.d.'s in cell parameters are only used when they are defined by crystal symmetry. An approximate (isotropic) treatment of cell e.s.d.'s is used for estimating e.s.d.'s involving l.s. planes.
Refinement. Refinement of *F*^2^ against ALL reflections. The weighted *R*-factor *wR* and goodness of fit *S* are based on *F*^2^, conventional *R*-factors *R* are based on *F*, with *F* set to zero for negative *F*^2^. The threshold expression of *F*^2^ > σ(*F*^2^) is used only for calculating *R*-factors(gt) *etc*. and is not relevant to the choice of reflections for refinement. *R*-factors based on *F*^2^ are statistically about twice as large as those based on *F*, and *R*- factors based on ALL data will be even larger.

### Fractional atomic coordinates and isotropic or equivalent isotropic displacement parameters (Å^2^)

**Table:**

	*x*	*y*	*z*	*U*_iso_*/*U*_eq_	Occ. (<1)
Pr1	0.0000	0.0000	0.0000	0.00947 (18)	0.685 (3)
Ca1	0.0000	0.0000	0.0000	0.00947 (18)	0.315 (3)
Mo1	0.5000	0.5000	0.5000	0.00539 (19)	
O1	0.4247 (3)	0.1250	0.1250	0.0160 (5)	
O2	0.1250	0.1250	0.1250	0.0100 (8)	

### Atomic displacement parameters (Å^2^)

**Table:**

	*U*^11^	*U*^22^	*U*^33^	*U*^12^	*U*^13^	*U*^23^
Pr1	0.00947 (18)	0.00947 (18)	0.00947 (18)	−0.00154 (5)	−0.00154 (5)	−0.00154 (5)
Ca1	0.00947 (18)	0.00947 (18)	0.00947 (18)	−0.00154 (5)	−0.00154 (5)	−0.00154 (5)
Mo1	0.00539 (19)	0.00539 (19)	0.00539 (19)	−0.00013 (5)	−0.00013 (5)	−0.00013 (5)
O1	0.0239 (14)	0.0120 (6)	0.0120 (6)	0.000	0.000	−0.0026 (8)
O2	0.0100 (8)	0.0100 (8)	0.0100 (8)	0.000	0.000	0.000

### Geometric parameters (Å, °)

**Table:**

Pr1—O2^i^	2.2588	Mo1—Ca1^xvii^	3.68859 (11)
Pr1—O2	2.2588	Mo1—Ca1^xviii^	3.68859 (11)
Pr1—O1^ii^	2.5930 (18)	Mo1—Ca1^xix^	3.68859 (11)
Pr1—O1^iii^	2.5930 (18)	Mo1—Ca1^xx^	3.68859 (11)
Pr1—O1^iv^	2.5930 (18)	Mo1—Ca1^xxi^	3.68859 (11)
Pr1—O1^v^	2.5930 (18)	Mo1—Ca1^xxii^	3.68859 (11)
Pr1—O1^vi^	2.5930 (18)	O1—Mo1^xxiii^	2.0046 (10)
Pr1—O1^vii^	2.5930 (18)	O1—Mo1^xxiv^	2.0046 (10)
Pr1—Pr1^vii^	3.68859 (11)	O1—Pr1^vii^	2.5930 (18)
Pr1—Ca1^viii^	3.68859 (11)	O1—Ca1^vii^	2.5930 (18)
Pr1—Ca1^ix^	3.68859 (11)	O1—Ca1^xxv^	2.5930 (18)
Pr1—Ca1^x^	3.68859 (11)	O1—Pr1^xxv^	2.5930 (18)
Mo1—O1^xi^	2.0046 (10)	O2—Ca1^xxv^	2.2588
Mo1—O1^xii^	2.0046 (10)	O2—Pr1^vii^	2.2588
Mo1—O1^xiii^	2.0046 (10)	O2—Pr1^ix^	2.2588
Mo1—O1^xiv^	2.0046 (10)	O2—Pr1^xxv^	2.2588
Mo1—O1^xv^	2.0046 (10)	O2—Ca1^vii^	2.2588
Mo1—O1^xvi^	2.0046 (10)	O2—Ca1^ix^	2.2588
			
O2^i^—Pr1—O2	180.0	O1^xii^—Mo1—Ca1^xvii^	42.51 (5)
O2^i^—Pr1—O1^ii^	79.93 (4)	O1^xiii^—Mo1—Ca1^xvii^	137.49 (5)
O2—Pr1—O1^ii^	100.07 (4)	O1^xiv^—Mo1—Ca1^xvii^	90.0
O2^i^—Pr1—O1^iii^	79.93 (4)	O1^xv^—Mo1—Ca1^xvii^	137.49 (5)
O2—Pr1—O1^iii^	100.07 (4)	O1^xvi^—Mo1—Ca1^xvii^	42.51 (5)
O1^ii^—Pr1—O1^iii^	117.01 (2)	O1^xi^—Mo1—Ca1^xviii^	42.51 (5)
O2^i^—Pr1—O1^iv^	79.93 (4)	O1^xii^—Mo1—Ca1^xviii^	90.0
O2—Pr1—O1^iv^	100.07 (4)	O1^xiii^—Mo1—Ca1^xviii^	137.49 (5)
O1^ii^—Pr1—O1^iv^	117.01 (2)	O1^xiv^—Mo1—Ca1^xviii^	137.49 (5)
O1^iii^—Pr1—O1^iv^	117.01 (2)	O1^xv^—Mo1—Ca1^xviii^	90.0
O2^i^—Pr1—O1^v^	100.07 (4)	O1^xvi^—Mo1—Ca1^xviii^	42.51 (5)
O2—Pr1—O1^v^	79.93 (4)	Ca1^xvii^—Mo1—Ca1^xviii^	60.0
O1^ii^—Pr1—O1^v^	180.00 (8)	O1^xi^—Mo1—Ca1^xix^	42.51 (5)
O1^iii^—Pr1—O1^v^	62.99 (2)	O1^xii^—Mo1—Ca1^xix^	137.49 (5)
O1^iv^—Pr1—O1^v^	62.99 (2)	O1^xiii^—Mo1—Ca1^xix^	90.0
O2^i^—Pr1—O1^vi^	100.07 (4)	O1^xiv^—Mo1—Ca1^xix^	137.49 (5)
O2—Pr1—O1^vi^	79.93 (4)	O1^xv^—Mo1—Ca1^xix^	42.51 (5)
O1^ii^—Pr1—O1^vi^	62.99 (2)	O1^xvi^—Mo1—Ca1^xix^	90.0
O1^iii^—Pr1—O1^vi^	180.00 (8)	Ca1^xvii^—Mo1—Ca1^xix^	120.0
O1^iv^—Pr1—O1^vi^	62.99 (2)	Ca1^xviii^—Mo1—Ca1^xix^	60.0
O1^v^—Pr1—O1^vi^	117.01 (2)	O1^xi^—Mo1—Ca1^xx^	137.49 (5)
O2^i^—Pr1—O1^vii^	100.07 (4)	O1^xii^—Mo1—Ca1^xx^	90.0
O2—Pr1—O1^vii^	79.93 (4)	O1^xiii^—Mo1—Ca1^xx^	42.51 (5)
O1^ii^—Pr1—O1^vii^	62.99 (2)	O1^xiv^—Mo1—Ca1^xx^	42.51 (5)
O1^iii^—Pr1—O1^vii^	62.99 (2)	O1^xv^—Mo1—Ca1^xx^	90.0
O1^iv^—Pr1—O1^vii^	180.00 (8)	O1^xvi^—Mo1—Ca1^xx^	137.49 (5)
O1^v^—Pr1—O1^vii^	117.01 (2)	Ca1^xvii^—Mo1—Ca1^xx^	120.0
O1^vi^—Pr1—O1^vii^	117.01 (2)	Ca1^xviii^—Mo1—Ca1^xx^	180.0
O2^i^—Pr1—Pr1^vii^	144.7	Ca1^xix^—Mo1—Ca1^xx^	120.0
O2—Pr1—Pr1^vii^	35.3	O1^xi^—Mo1—Ca1^xxi^	90.0
O1^ii^—Pr1—Pr1^vii^	135.34 (4)	O1^xii^—Mo1—Ca1^xxi^	137.49 (5)
O1^iii^—Pr1—Pr1^vii^	81.87 (4)	O1^xiii^—Mo1—Ca1^xxi^	42.51 (5)
O1^iv^—Pr1—Pr1^vii^	81.87 (4)	O1^xiv^—Mo1—Ca1^xxi^	90.0
O1^v^—Pr1—Pr1^vii^	44.66 (4)	O1^xv^—Mo1—Ca1^xxi^	42.51 (5)
O1^vi^—Pr1—Pr1^vii^	98.13 (4)	O1^xvi^—Mo1—Ca1^xxi^	137.49 (5)
O1^vii^—Pr1—Pr1^vii^	98.13 (4)	Ca1^xvii^—Mo1—Ca1^xxi^	180.0
O2^i^—Pr1—Ca1^viii^	35.3	Ca1^xviii^—Mo1—Ca1^xxi^	120.0
O2—Pr1—Ca1^viii^	144.7	Ca1^xix^—Mo1—Ca1^xxi^	60.0
O1^ii^—Pr1—Ca1^viii^	44.66 (4)	Ca1^xx^—Mo1—Ca1^xxi^	60.0
O1^iii^—Pr1—Ca1^viii^	98.13 (4)	O1^xi^—Mo1—Ca1^xxii^	137.49 (5)
O1^iv^—Pr1—Ca1^viii^	98.13 (4)	O1^xii^—Mo1—Ca1^xxii^	42.51 (5)
O1^v^—Pr1—Ca1^viii^	135.34 (4)	O1^xiii^—Mo1—Ca1^xxii^	90.0
O1^vi^—Pr1—Ca1^viii^	81.87 (4)	O1^xiv^—Mo1—Ca1^xxii^	42.51 (5)
O1^vii^—Pr1—Ca1^viii^	81.87 (4)	O1^xv^—Mo1—Ca1^xxii^	137.49 (5)
Pr1^vii^—Pr1—Ca1^viii^	180.0	O1^xvi^—Mo1—Ca1^xxii^	90.0
O2^i^—Pr1—Ca1^ix^	144.7	Ca1^xvii^—Mo1—Ca1^xxii^	60.0
O2—Pr1—Ca1^ix^	35.3	Ca1^xviii^—Mo1—Ca1^xxii^	120.0
O1^ii^—Pr1—Ca1^ix^	81.87 (4)	Ca1^xix^—Mo1—Ca1^xxii^	180.0
O1^iii^—Pr1—Ca1^ix^	81.87 (4)	Ca1^xx^—Mo1—Ca1^xxii^	60.0
O1^iv^—Pr1—Ca1^ix^	135.34 (4)	Ca1^xxi^—Mo1—Ca1^xxii^	120.0
O1^v^—Pr1—Ca1^ix^	98.13 (4)	Mo1^xxiii^—O1—Mo1^xxiv^	133.86 (14)
O1^vi^—Pr1—Ca1^ix^	98.13 (4)	Mo1^xxiii^—O1—Pr1^vii^	105.99 (3)
O1^vii^—Pr1—Ca1^ix^	44.66 (4)	Mo1^xxiv^—O1—Pr1^vii^	105.99 (3)
Pr1^vii^—Pr1—Ca1^ix^	60.0	Mo1^xxiii^—O1—Ca1^vii^	105.99 (3)
Ca1^viii^—Pr1—Ca1^ix^	120.0	Mo1^xxiv^—O1—Ca1^vii^	105.99 (3)
O2^i^—Pr1—Ca1^x^	35.3	Mo1^xxiii^—O1—Ca1^xxv^	105.99 (3)
O2—Pr1—Ca1^x^	144.7	Mo1^xxiv^—O1—Ca1^xxv^	105.99 (3)
O1^ii^—Pr1—Ca1^x^	98.13 (4)	Pr1^vii^—O1—Ca1^xxv^	90.68 (8)
O1^iii^—Pr1—Ca1^x^	98.13 (4)	Ca1^vii^—O1—Ca1^xxv^	90.68 (8)
O1^iv^—Pr1—Ca1^x^	44.66 (4)	Mo1^xxiii^—O1—Pr1^xxv^	105.99 (3)
O1^v^—Pr1—Ca1^x^	81.87 (4)	Mo1^xxiv^—O1—Pr1^xxv^	105.99 (3)
O1^vi^—Pr1—Ca1^x^	81.87 (4)	Pr1^vii^—O1—Pr1^xxv^	90.68 (8)
O1^vii^—Pr1—Ca1^x^	135.34 (4)	Ca1^vii^—O1—Pr1^xxv^	90.68 (8)
Pr1^vii^—Pr1—Ca1^x^	120.0	Pr1—O2—Ca1^xxv^	109.5
Ca1^viii^—Pr1—Ca1^x^	60.0	Pr1—O2—Pr1^vii^	109.5
Ca1^ix^—Pr1—Ca1^x^	180.0	Ca1^xxv^—O2—Pr1^vii^	109.5
O1^xi^—Mo1—O1^xii^	94.97 (9)	Pr1—O2—Pr1^ix^	109.5
O1^xi^—Mo1—O1^xiii^	94.97 (9)	Ca1^xxv^—O2—Pr1^ix^	109.5
O1^xii^—Mo1—O1^xiii^	94.97 (9)	Pr1^vii^—O2—Pr1^ix^	109.5
O1^xi^—Mo1—O1^xiv^	180.0	Pr1—O2—Pr1^xxv^	109.5
O1^xii^—Mo1—O1^xiv^	85.03 (9)	Pr1^vii^—O2—Pr1^xxv^	109.5
O1^xiii^—Mo1—O1^xiv^	85.03 (9)	Pr1^ix^—O2—Pr1^xxv^	109.5
O1^xi^—Mo1—O1^xv^	85.03 (9)	Pr1—O2—Ca1^vii^	109.5
O1^xii^—Mo1—O1^xv^	180.0	Ca1^xxv^—O2—Ca1^vii^	109.5
O1^xiii^—Mo1—O1^xv^	85.03 (9)	Pr1^ix^—O2—Ca1^vii^	109.5
O1^xiv^—Mo1—O1^xv^	94.97 (9)	Pr1^xxv^—O2—Ca1^vii^	109.5
O1^xi^—Mo1—O1^xvi^	85.03 (9)	Pr1—O2—Ca1^ix^	109.5
O1^xii^—Mo1—O1^xvi^	85.03 (9)	Ca1^xxv^—O2—Ca1^ix^	109.5
O1^xiii^—Mo1—O1^xvi^	180.0	Pr1^vii^—O2—Ca1^ix^	109.5
O1^xiv^—Mo1—O1^xvi^	94.97 (9)	Pr1^xxv^—O2—Ca1^ix^	109.5
O1^xv^—Mo1—O1^xvi^	94.97 (9)	Ca1^vii^—O2—Ca1^ix^	109.5
O1^xi^—Mo1—Ca1^xvii^	90.0		

Symmetry codes: (i) −*x*, −*y*, −*z*; (ii) −*y*, *z*−1/4, *x*−1/4; (iii) *z*−1/4, *x*−1/4, −*y*; (iv) *x*−1/4, *y*−1/4, −*z*; (v) *y*, −*z*+1/4, −*x*+1/4; (vi) −*z*+1/4, −*x*+1/4, *y*; (vii) −*x*+1/4, −*y*+1/4, *z*; (viii) −*x*−1/4, −*y*−1/4, *z*; (ix) *x*, −*y*+1/4, −*z*+1/4; (x) *x*, −*y*−1/4, −*z*−1/4; (xi) −*y*+1/2, −*z*+1/2, −*x*+1; (xii) −*z*+1/2, −*x*+1, −*y*+1/2; (xiii) −*x*+1, −*y*+1/2, −*z*+1/2; (xiv) *y*+1/2, *z*+1/2, *x*; (xv) *z*+1/2, *x*, *y*+1/2; (xvi) *x*, *y*+1/2, *z*+1/2; (xvii) −*x*+1/4, −*y*+3/4, *z*+1/2; (xviii) *y*+1/4, −*x*+1/2, *z*+3/4; (xix) *x*+1/2, −*y*+1/4, −*z*+3/4; (xx) *y*+3/4, −*x*+1/2, *z*+1/4; (xxi) −*x*+3/4, −*y*+1/4, *z*+1/2; (xxii) *x*+1/2, −*y*+3/4, −*z*+1/4; (xxiii) *x*, −*y*+3/4, −*z*+3/4; (xxiv) *x*, *y*−1/2, *z*−1/2; (xxv) *y*+1/4, −*x*, *z*+1/4.
